# In Vitro Evaluation of the Influence of Biosynthesized Calcium Oxide Nanoparticles on the Antibacterial Activity, pH, Microleakage and Cytotoxicity of Conventional Intracanal Medicaments

**DOI:** 10.3390/ijms252211991

**Published:** 2024-11-08

**Authors:** Fasiha Moin Kazi, Khurram Parvez, Asif Asghar, Shazia Akbar, Noor-ul-Ain Jawaed, Naresh Kumar, Paulo J. Palma

**Affiliations:** 1 Department of Science of Dental Materials, Dow Dental College, Dow University of Health Sciences, Karachi 74200, Pakistan; kumar.naresh@duhs.edu.pk; 2 Science of Dental Materials, Dr. Ishrat ul Ebad Khan Institute of Oral Health Sciences, Dow University of Health Sciences, Karachi 74200, Pakistan; khurram.parvez@duhs.edu.pk; 3 Food and Feed Safety, Pakistan Council for Scientific and Industrial Research Laboratories, Karachi 75280, Pakistan; masif345@yahoo.com; 4 Department of Oral Pathology, Dow Dental College, Dow University of Health Sciences, Karachi 74200, Pakistan; shazia.akbar@duhs.edu.pk; 5 Department of Operative Dentistry, Dr. Ishrat ul Ebad Khan Institute of Oral Health Sciences, Dow University of Health Sciences, Karachi 74200, Pakistan; noorulain.jawaed@duhs.edu.pk; 6 Center for Innovation and Research in Oral Sciences (CIROS), Faculty of Medicine, University of Coimbra, 3000-075 Coimbra, Portugal; 7 Institute of Endodontics, Faculty of Medicine, University of Coimbra, 3000-075 Coimbra, Portugal

**Keywords:** biosynthesis, calcium oxide, endodontics, *Enterococcus faecalis*, intracanal medicaments, nanoparticles

## Abstract

Intracanal medicaments are an important adjunct to the effective disinfection of the root canal system. However, conventional intracanal medicaments do not provide adequate protection against *Enterococcus faecalis*, which is the organism of interest in many cases of root canal failures. This study aimed to evaluate the influence of biosynthesized calcium oxide nanoparticles (CaO NPs) on the antibacterial activity, pH, microleakage and cytotoxicity of intracanal medicaments. CaO NPs were biosynthesized by the direct thermal decomposition of eggshells (EGS) and the reduction of calcium nitrate with papaya leaf extract (PLE). These nanoparticles were mixed with a proprietary calcium hydroxide powder in 10% and 25% (*w*/*w*) concentrations and blended in analytical-grade coconut oil to formulate the experimental medicaments. These were then evaluated for antibacterial activity, pH, microleakage and cytotoxicity at 1 day, 7 days and 15 days. A proprietary calcium hydroxide paste formulation (MX) was used as the control. Means and standard deviations were calculated and analyzed using repeated-measures ANOVA for pH and three-way ANOVA for the antibacterial effect, microleakage and cytotoxicity, followed by LSD post hoc analysis. Significant antibacterial activity was noted against *Enterococcus faecalis* at all times, with zones of inhibition (ZOI) up to 19.60 ± 2.30 mm. pH levels up to 13.13 ± 0.35 were observed for the experimental groups. Microleakage remained comparable to the control, while cytotoxicity was not observed in any of the groups at any time. Intracanal medicaments formulated with 10% and 25% (*w*/*w*) of biosynthesized CaO NPs could be promising candidates for the disinfection of the root canal system compared to conventional counterparts.

## 1. Introduction

Infections of endodontic origin can be broadly categorized into primary infections, secondary and/or persistent infections. *Enterococcus faecalis* is predominantly associated with secondary or persistent infections of the root canal system [1]. Secondary and persistent infections account for most flare-ups during treatment and unresolved apical periodontitis, leading to root canal failures. Treatment protocol varies with the severity of the infection and includes a complete debridement and disinfection of the root canal system, aided with intracanal medicaments and systemic antibiotics [2].

Due to the emergence of resistant strains and a worldwide awareness of antibiotic resistance, the focus has shifted to improving the efficacy of local drug delivery systems. The standard intracanal medicaments are based on calcium hydroxide, which is a powerful antibacterial agent, effective against most Gram-positive and -negative bacteria [3,4]. Calcium hydroxide acts mainly by creating an alkaline pH that disrupts the bacterial structure, leading to cell lysis [5]. However, the efficacy of calcium hydroxide against *Enterococcus faecalis* remains questionable [1,6,7]. Hence, there is a need to enhance the efficacy of intracanal medicaments with the incorporation of additives which can eradicate *Enterococcus faecalis* effectively from the root canal system. The investigation of new medicaments or compounds that provide this effect could add value to the current body of literature which deals with endodontic disinfection.

Metal oxide nanoparticles (NPs) have been shown to demonstrate superior antimicrobial properties [7,8]. Given the fact that NPs act along multiple cellular pathways simultaneously, the ability of the organism to develop resistance against their activity is greatly reduced [8,9]. Thus, researchers have acknowledged the role of antimicrobial NPs in combatting antibiotic resistance in microbes [10,11]. Likewise, calcium oxide (CaO) NPs were found to be effective in a variety of biomedical applications. These included their use as adsorbents for heavy metal removal from water [12], as heterogeneous catalysts in biodiesel production [12], in clinical translation for cancer therapy, as well as antibacterial agents in endodontic sealers [13]. Calcium oxide in the regular form was already being used in several proprietary dental products e.g., MTA Angelus^®^, Biodentine^TM^, EndoCal 10^®^ and BioC-Repair^®^ [14]. Given the chemical compatibility with calcium hydroxide, the nano form of calcium oxide could be easily incorporated into intracanal medicaments to enhance the antibacterial effect.

The synthesis of NPs by conventional methods involves sophisticated equipment, hazardous chemicals and by-products, which has led researchers to opt for green chemistry-based biosynthesis techniques [12]. Green chemistry as a field of science was introduced by the Environmental Protection Agency in the 1980s to promote safer chemical processes that reduce damage to the environment [15]. Thus, the literature describes the biosynthesis of nanoparticles using agro-wastes and plant extracts as raw materials, along with reducing and capping agents [16].

This study aimed to explore the influence of intracanal medicaments based on biosynthesized CaO NPs on antibacterial activity, pH, microleakage and cytotoxicity. These properties had a significant bearing on the activity and physiochemical characteristics of the medicaments [1]. The antibacterial activity of CaO and hydroxide-based intracanal pastes was primarily governed by the level of alkalinity produced [1,5]. It was reported in the literature that *Enterococcus faecalis* was able to sustain pH levels up to 11.5 approximately [17,18], so a higher and sustained level of alkalinity was required to combat the bacterium. This could be achieved if the intracanal paste produced a steady rate of dissociation yet remained within the confines of the canal system for the designated treatment duration. This implied minimal or no leaching out in the periapical tissues, resulting in low levels of microleakage. As intracanal medicaments remained in the canal system for almost fifteen days on average, it was imperative that they did not produce any cytotoxic effects on the dental and periodontal tissues.

The null hypothesis tested was that the experimental medicaments would not demonstrate antibacterial activity against *Enterococcus faecalis*, nor produce high pH and microleakage as well, and cytotoxicity would increase significantly when compared to the proprietary control.

## 2. Results

### 2.1. Characterization

**Figure 1:**
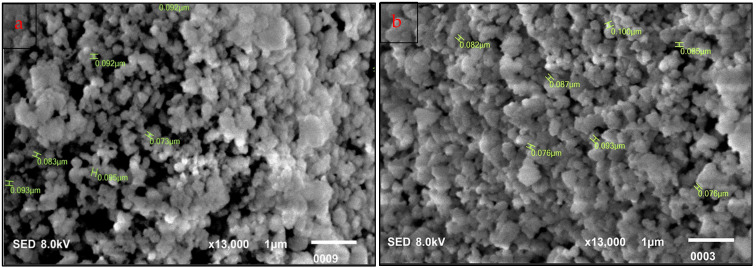
Scanning electron microscopy images for (**a**) EGS and (**b**) PLE calcium oxide nanoparticles powder samples before the formulation of the intracanal pastes. Spherical and quasi-spherical calcium oxide nanoparticles are visible, arranged in clusters with particle sizes marked in green at 13,000× magnification. Particle sizes measured by image analysis software pertaining to JSM IT100 (JEOL Ltd., Tokyo, Japan).

SEM images showed that the nanoparticles were spherical or quasi-spherical, arranged in agglomerates, with a mean diameter of 81nm (approx.) for EGS and 85 nm (approx.) for the PLE group (Figure 1).

**Figure 2:**
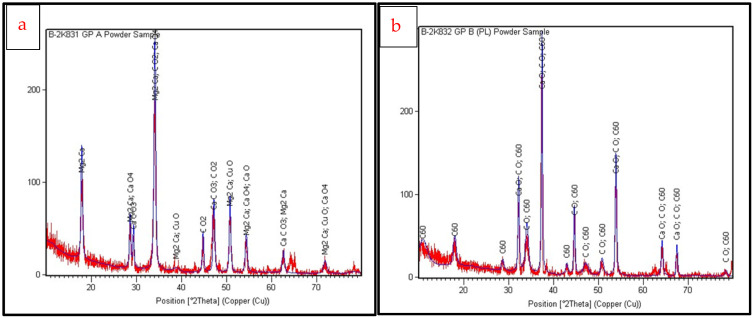
X-ray diffractograms for the nanoparticle samples of (**a**) EGS and (**b**) PLE calcium oxide nanoparticles in powder form showing the typical peaks observed for CaO and other biosynthesis derivatives. Images obtained with image analysis software pertaining to XPERT-PRO 11063168, 30 mA and 40 kV.

The diffraction data and cubic structure of the crystallites were in accordance with ICDD Ref. No. 00-037-1497 for calcium oxide. The crystallites of CaOe were cubic in structure, with a mean size of 18.97 nm and a phase percentage of 27% for EGS, whereas a mean size of 24.89 nm and a phase percentage of 32% was observed for the PLE samples. Traces of magnesium, carbon, etc., could also be detected, suggesting the presence of biosynthesis derivatives (Figure 2).

### 2.2. Antibacterial Activity Evaluation with Agar Diffusion Assay

**Figure 3:**
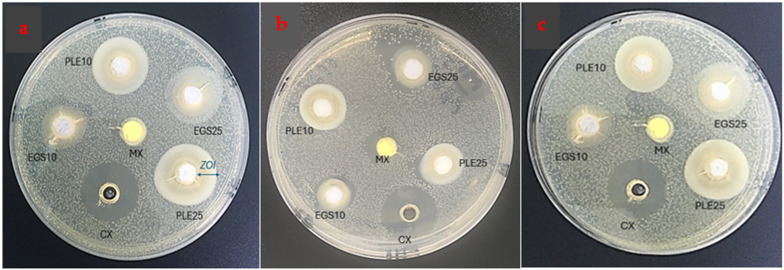
The antibacterial activity of the intracanal medicaments (intra-experiment) as evaluated by the agar well diffusion assay. (**a**). After 1 day of incubation. (**b**). After 7 days of incubation. (**c**). After 15 days of incubation. Zones of inhibition surrounding each medicament can be observed and measured as marked with arrows in a. The difference in the dimensions of the inhibition zone of the proprietary control (MX) and the experimental medicaments can be appreciated. Image captured by the author using an iPhone SE (2023), edited with ‘Microsoft Photos’ (version 2024.11070.11002.0).

**Figure 4:**
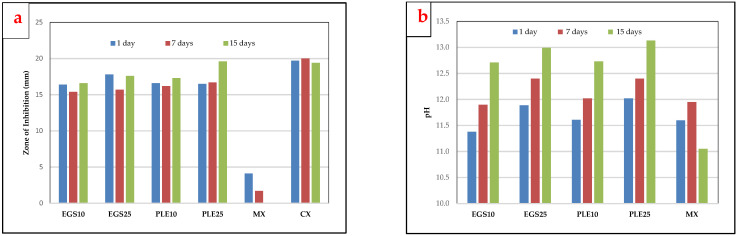

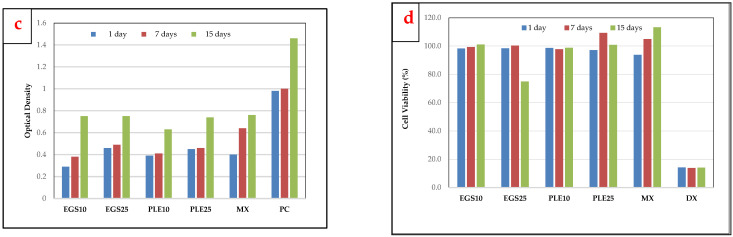
Graphical representation of the results. (**a**). Antibacterial activity against *Enterococcus faecalis* evaluated with the agar well diffusion assay; (**b**). pH evaluation; (**c**). microleakage evaluation with the dye extraction method; (**d**). cytotoxicity assessment with the MTT Assay.

The experimental medicaments were able to demonstrate antibacterial activity against *Enterococcus faecalis* (Table 1, Figure 3 and Figure 4). On the contrary, negligible activity was observed for the proprietary control (MX). The inhibition zones for the experimental medicaments were significantly larger than MX at all times (*p* < 0.001) and were comparable to 0.2% chlorhexidine digluconate (CX) as positive control after 1 day and 15 days. There was no significant difference in the antibacterial activity of the EGS medicaments and the PLE medicaments. The ZOI values obtained for the 25% concentration were higher than the 10% concentration, but this difference was not statistically significant.

### 2.3. pH Evaluation

There was a steady rise in the pH for the experimental medicaments from 1 day to 15 days, unlike the proprietary control (MX), for which pH decreased in this duration (Table 2 and Figure 4). This denotes that the degree of dissociation remained steady for the experimental medicaments throughout the 15-day period, resulting in a continuous supply of hydroxyl ions. On the contrary, the hydroxyl ion release for the proprietary medicament was exhausted after 7 days. There was no significant difference in the pH of EGS and PLE medicaments as a whole. However, pH values for the 25% NP medicaments were significantly higher than the 10% NP medicaments at all time intervals (*p* < 0.001). The highest pH was observed for PLE25 after 15 days.

### 2.4. Microleakage Evaluation

No significant differences were observed in microleakage between the experimental medicaments and the proprietary control MX (Table 3 and Figure 4) at any time (0.11 ≤ *p* ≤ 0.91). Also, no significant difference was observed between EGS and PLE medicaments at any time (0.54 ≤ *p* ≤ 1.00). All the medicaments demonstrated significantly lower microleakage than the unfilled roots that were taken as the positive control (PC) at all times (*p* < 0.001). This shows that the microleakage for the experimental groups was not affected adversely by the nano form of calcium oxide and remained within acceptable limits.

### 2.5. Cytotoxicity

**Figure 5:**
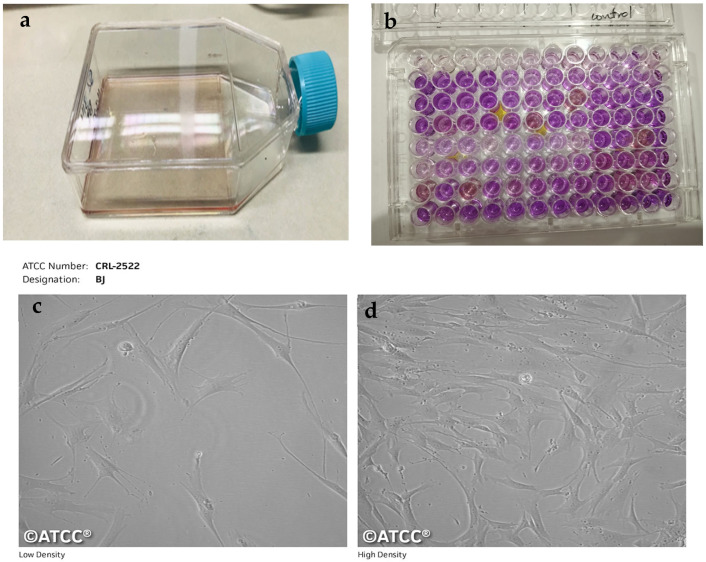
BJ cell line human fibroblasts (**a**). cultured in the flask (intra-experiment), (**b**). in a 96-well plate for cytotoxicity evaluation with the MTT Assay (intra-experiment). (**c**,**d**) Pre-experiment BJ cell line fibroblasts as observed under a microscope. Cells were obtained from ATCC (CRL-2522). Image (**c**,**d**) courtesy: ATCC, https://www.atcc.org/products/crl-2522#product-references (accessed on 27 October 2024).

Cell viability data (Table 4) showed that all the intracanal medicaments, experimental and control, were non-cytotoxic to the fibroblast cell line (Figure 5). Cell viability higher than 72% was observed at all time intervals. A proliferative effect was observed for EGS10, EGS25, PLE25 and MX after 7 days and/or 15 days of incubation. Significant differences were observed with the cytotoxic drug doxorubicin (DX) at all time intervals (*p* < 0.001).

## 3. Discussion

The effective biosynthesis of CaO NPs in this study corroborates the findings in the literature where agro-waste and plant sources were used [19,20,21]. Habte L et al. [12] reported the synthesis of CaO NPs using waste eggshells, which were “almost spherical” in morphology, agglomerated, with a mean diameter of 198 nm. Singh A et al. [9] described how phytochemicals acted as capping and reducing agents to control nanoparticle size and morphology.

Biosynthesized CaO NPs have multiple scientific and industrial applications. Potent antibacterial and antioxidant effects make these suitable for localized biological applications, such as in the root canal system [8,22]. Also, NPs target different biomolecules simultaneously; multiple pathways are employed, making it difficult for bacteria to develop resistance against them. Thus, the rampant use of systemic antibiotics, which contributes to microbial resistance, can be avoided. Dewi et al. [23] also discussed that antibiotics used in high concentrations diffused to the cementodentinal junction, resulting in drug allergy and accumulation. On the other hand, drug-resistant strains developed if the concentration of the drug was not high enough to eliminate the bacteria. Conwell et al. [24] describe the inclusion of enterococci in the WHO list of priority pathogens which are resistant to multiple antibiotics. In particular, *Enterococcus faecalis* was predominantly associated with secondary or persistent infections of the root canal system. This led to an increase in clinical visits and a greater incidence of flare-ups and treatment failures.

The results of this study support the use of CaO NPs against *Enterococcus faecalis*. The experimental intracanal pastes demonstrated a significant antimicrobial effect. This was in sharp contrast to the proprietary control containing calcium hydroxide, iodoform and silicon oil, which showed negligible activity against *Enterococcus faecalis*. The activity of PLE medicament was found to be comparable to 0.2% chlorhexidine digluconate, which was used as a positive control. The application of chlorhexidine as an adjunct to calcium hydroxide was a practice followed by many clinicians. However, the results of this treatment appeared contradictory in studies. Some authors report that chlorhexidine used at a 4% concentration was increasingly effective than at concentrations of 0.5%, 1% and 2%, proving that this effect was dose-dependent [7,25]. However, concerns about chlorhexidine toxicity, the degradation of chlorhexidine in alkaline environments and the production of parachloroanaline warranted a cautious use in biological tissues [1].

The fact that CaO NPs-based intracanal medicament could eliminate *Enterococcus faecalis* significantly better than a widely used commercial formulation corroborates with the results of other authors. Louwakul et al. [26] evaluated the activity of CaO NPs against *Enterococcus faecalis* by treating dentin specimens with fluorescent staining and then observing the number and viability of the bacterial cells. The authors concluded that CaO NPs destroyed significantly more bacteria than the regular form of calcium oxide or calcium hydroxide. Ahmad et al. [19] discussed the antibacterial effects of biosynthesized CaO NPs against a variety of microbes and observed significant antibacterial activity (ZOI up to 26.67 ± 0.5 mm). Similar results were reported in the literature by using CaO NPs synthesized with chicken eggshells as a source of calcium carbonate [12,27].

The use of agar diffusion assay for initial antibiotic susceptibility testing was a standard practice for many researchers. The assay allows for the simultaneous evaluation of multiple test substances against a single microorganism, enabling comparative analysis of their antimicrobial activity [22]. It provided a simple, reproducible and cost-effective method and was applicable to a broad range of antibiotics and bacterial species. However, the results were dependent on a number of factors, like the diffusion of the antibiotic through the agar and the growth of the bacterium on the agar. Also, it did not offer an evaluation of the minimum inhibitory concentration which was required for more tailored treatment protocols [22].

In this study, the steady rise in pH in the experimental groups pointed towards effective dissociation of the medicament over a sustained period of time, leading to effective disinfection [3,19]. The coconut oil vehicle provided the right consistency and allowed a continuous release of ions to increase the pH [28,29]. This was contradictory to the control group, where the pH remained lower, and a sharp decline was observed from 7 days to 15 days. Coconut oil mixed well with the calcium oxide powder and helped in achieving the right consistency for the paste [30]. Moreover, it did not adversely affect the dissolution of the paste from the canal, which was evident from the microleakage results. The leaching of the experimental medicaments over time was similar to the control [31]. This was in contrast to the studies on NPs which report a greater dissolution and resultant microleakage at nanoscale [31]. Here, the role of additives and vehicles becomes important, as thickening agents and viscous vehicles can help control microleakage. Thus, in this study, regular calcium hydroxide powder was used as a thickening agent and coconut oil as a vehicle.

The dye extraction method used in the study was a more reliable indicator of microleakage than linear dye penetration as it provided a volumetric measurement of the dye penetration in the specimen. It also allowed quantitative analysis by spectrophotometry, enabling reproducibility and objectivity. Compared to the fluid filtration method, it was a simple procedure that did not require complex equipment. And unlike the fluid filtration method, where the filtration values tend to diminish over time, leading to a plateau when all irregularities are penetrated by the water, the dye extraction method provided a more consistent and reliable result [32]. However, in vitro microleakage studies have their limitations in terms of their inability to replicate actual clinical conditions where pH, temperature fluctuations and mechanical forces come into play affecting microleakage. Low molecular weight dyes like methylene blue can penetrate areas where there is not clinically significant leakage, resulting in a potential overestimation of microleakage. However, air entrapped inside root canals may also hinder fluid movement [32].

Cytotoxicity levels remained negligible at all time intervals for all the groups of intracanal medicaments. This was in line with studies where other calcium hydroxide-based formulations were tested, and more than 70% of cell viability levels were observed for human gingival fibroblasts [33]. In this study, proliferative activity was also observed in many of the samples after 7 days and 15 days.

The use of the BJ cell line, which was based on human fibroblasts, was more suitable for studies requiring human cellular responses, e.g., testing the cytotoxicity of the medicaments in the human body as compared to the L929 cell line derived from mice. Also, the BJ cell line was derived from normal human fibroblasts, while the L929 was a transformed cell line (cells showed mutations that allow indefinite division). Consequently, BJ cells better represent primary fibroblasts with similar growth rates, morphology and gene expression. However, given the technique sensitivity of the procedure, it was difficult to achieve precise results after prolonged intervals, so it would be more accurate to restrict ourselves to the findings after 24 h.

Overall, the study encompassed the most important aspects of intracanal medicaments like the antibacterial activity, pH, microleakage and cytotoxicity evaluation. Another strength was the biosynthesis of CaO NPs, which avoided the use of hazardous reagents, catalysts and processes that would be damaging to the personnel and the environment. The medicament thus produced exhibited satisfactory physicochemical characteristics comparable to the commercial calcium hydroxide-based control. Thus, the null hypothesis was rejected. Also, it superseded the control in terms of pH and antimicrobial efficacy and produced a sustained effect throughout the duration of the study.

However, this was a preliminary study on the role of CaO NPs in intracanal medicaments. Further studies were required to explore other aspects of the activity of intracanal medicaments, e.g., to ascertain the depth of penetration of the medicament in the dentinal tubules. The biocompatibility of the medicament also warranted a more detailed analysis with measurement of inflammatory and sensitization responses. The investigation of antibacterial activity by contemporary analytical techniques like confocal laser scanning microscopy and the utilization of primary cultures and multispecies biofilms could not be carried out because of resource constraints.

## 4. Materials and Methods

This study was carried out after obtaining approval from the Institutional Review Board of the Dow University of Health Sciences, Karachi, Pakistan (IRB-1871/DUHS/Approval/2020).

### 4.1. Synthesis of Calcium Oxide Nanoparticles

Two different techniques were adopted for the biosynthesis of NPs. One method utilized eggshells (Table 5) as a source of calcium carbonate, which was converted into CaO NPs via direct thermal decomposition [30,34]. The other method involved the conversion of calcium nitrate to CaO NPs using papaya leaf extract as a reducing agent [20,35].

#### 4.1.1. Direct Thermal Decomposition of Eggshells (EGS)

Fifty chicken eggshells (194.58 g, obtained from regular broiler chicken, with smooth, untarnished, unstained and undamaged appearance) were collected from household eggshell waste, washed thoroughly with distilled water and then boiled for 15 min to eliminate microbes. The shells were left in the water to facilitate the removal of the inner protein layer using fine tweezers and dried in sunlight for 12 h. Thereafter, these were crushed into small pieces using a mortar and pestle and heated in a laboratory muffle furnace (LM-412, Linn High Therm GmbH, Hirschbach, Germany) to 900 °C for 1 h. At this stage, the shells appeared chalky white and brittle due to the conversion of calcium carbonate to calcium oxide. The shells were weighed again on the digital scale and found to weigh 145.52 g, indicating a weight loss of 49.06 g due to their decomposition into calcium oxide, liberating carbon dioxide.

CaCO_3_(s) → CaO(s) + CO_2_(g)

Some of the shells appeared burnt out, probably due to the remnants of the adherent inner protein layer on their surface and these were removed from the sample. This loss of eggshells amounted to a wastage of 96.72 g. The remainder shells were ground in a laboratory grinder (Foss Tecator 1093 Cyclotec Sample Mill, Foss Analytical AB, Höganäs, Sweden) for 3 min until a very fine powder was obtained. This was further sieved with a # 200 mesh. The resulting CaO powder weighed 48.8 g on a digital laboratory weighing scale. The CaO nanopowder thus obtained was stored in a screw-capped airtight container and placed in a desiccator until use.

#### 4.1.2. Reduction of Calcium Nitrate with Papaya Leaf Extract (PLE)

Papaya leaf extract was prepared from 100 g of thoroughly washed fresh papaya leaves. The leaves were immersed in 200 mL of distilled water and gradually heated to 100 °C, at which the mixture was allowed to boil for thirty minutes. Afterwards, the solution was cooled to room temperature and filtered with Whatman no. 40 filter paper to obtain a clear, green-coloured extract.

The calcium nitrate solution (1 M) was prepared by adding 23.6 g of calcium nitrate tetrahydrate powder (Table 5) to 100 mL of distilled water. An amount of 100 mL of papaya extract was added gradually to 100 mL of calcium nitrate solution (1:1). The mixture was stirred on a magnetic stirrer (Jenway 1000, Cole-Parmer Ltd., Stone, Staffordshire, UK) at 50 °C for about one hour. This changed the mixture from a clear yellowish green to a pale yellow with a milky appearance, confirming the reduction of calcium nitrate by the plant extract. The reaction between the calcium ions and the papaya leaf extract could be expressed as

nCa^2+^ + 2[Ar-C_6_H_3_(OH)_2_]n → nCaO + 2n(Ar-C_6_H_3_O_2_) + 2nH^+^,

where Ar-C_6_H_3_(OH)_2_ shows the aromatic ring of diphenols present in the plant extract, Ar-C_6_H_3_O_2_ is a quinone resulting from the oxidation of phenol, and n is the number of groups oxidized by metal ions.

Sodium hydroxide solution (1M) was prepared with 4 g of sodium hydroxide pellets (Table 5) and added to 100 mL distilled water. This solution (50 mL) was added dropwise to the calcium nitrate and papaya leaf mixture. Sodium hydroxide solution helped create an alkaline pH, thus encouraging precipitation of the nanoparticles. Stirring was continued till a yellow-white frothy precipitate was obtained.

Filtration was performed using Whatmann no. 40 filter paper. The precipitate was washed thoroughly with distilled water to remove the basicity of the solution. It was then dried (Memmert Dry Air Oven, Tv-15-20948, Memmert GmbH + Co. KG, Schwabach, Germany) at 105 °C for 3 h. Further, calcination was performed in a muffle furnace (LM-412, Linn High Therm GmbH, Germany) at 900 °C for one hour. The resultant material showed a change from yellow to chalky gray-white coarse powder. It was ground to a fine powder in a laboratory grinder for 3 min and passed through a #200 mesh sieve. The nanopowder obtained was weighed and found to be 7 g. Storage was performed in a screw-capped airtight container, placed in a desiccator until use.

### 4.2. Characterization

The morphology and size of nanoparticles were studied using scanning electron microscopy (SEM). Multiple images of up to 13,000 × magnification were scanned with the built-in automated image analysis software (JSM IT100, JEOL Ltd., Japan). A total of 100 nanoparticles were scanned for diameter by pointing the cursor at the particle of interest, and the readings were used to calculate the mean diameter as per the formula

Dm=Σd1−100N

where D_m_ is the mean diameter of the nanoparticles (approx.), Σd_1−100_ is the sum of the diameters of 100 particles, and N is the total number of particles.

The crystal structural analysis was analyzed by X-ray diffraction (XRD), with diffraction angles 2θ ranging from 10° to 80° and Cu Kα radiation (λ = 1.5406 Å) as the radiation source (XPERT-PRO 11063168, 30 mA and 40 kV). Automated software (XRD Crystallite/grain Size Calculator-Scherer Equation-InstaNANO. https://instanano.com/all/characterization/xrd/crystallite-size (accessed on 27 October 2024)) was used to calculate the mean crystallite size of the calcium oxide nanoparticles from the data obtained.

### 4.3. Formulation of Intracanal Medicaments

The NPs were mixed as 10% and 25% (*w*/*w*) with regular calcium hydroxide powder. The powder component was then mixed with analytical-grade coconut oil (Table 5), such that a thick, creamy consistency comparable to the proprietary formulation was achieved. The pastes were placed in Eppendorf tubes and labelled as EGS10, EGS25, PLE10 and PLE25 where EGS10: intracanal paste containing 10% (*w*/*w*) CaO NPs prepared by direct thermal decomposition of eggshells; EGS25: intracanal paste containing 25% (*w*/*w*) CaO NPs prepared by direct thermal decomposition of eggshells; PLE10: intracanal paste containing 10% (*w*/*w*) CaO NPs prepared by calcium nitrate reduction with papaya leaf extract; PLE25: intracanal paste containing 25% (*w*/*w*) CaO NPs prepared by calcium nitrate reduction with papaya leaf extract; MX: a widely used, calcium hydroxide based proprietary intracanal medicament (Metapex^®^, Meta Biomed Co., Ltd., Korea), was taken as control. It is composed of (approximate wt.%) 40% calcium hydroxide, 40% iodoform and 30% polydimethylsiloxane/silicon oil (Material Safety Data Sheet for Metapex Plus^®^). Iodoform is used to augment the antibacterial effect of calcium hydroxide. It also imparts radiopacity to the paste. Polydimethylsiloxane/silicon oil is used as an oily vehicle for the medicament to allow the sustained release of active ingredients.

The medicaments were analyzed for antibacterial activity against *Enterococcus faecalis*, pH, microleakage and cytotoxicity following sample size evaluation for each analysis.

### 4.4. Sample Size Calculation

**Figure 6:**
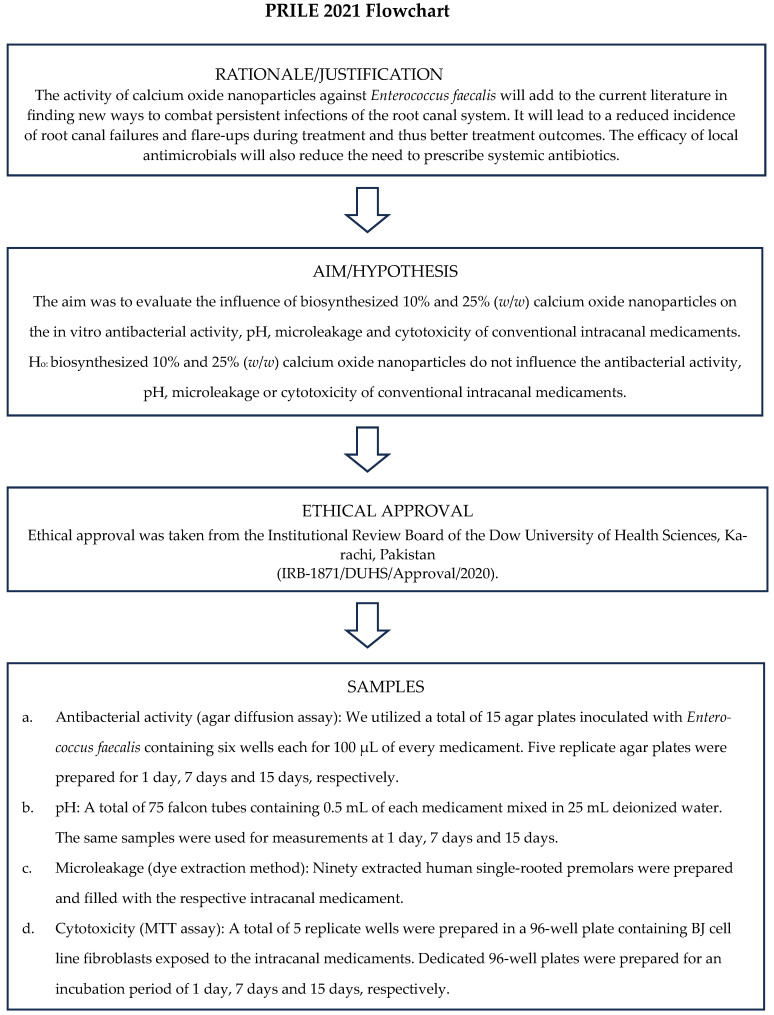

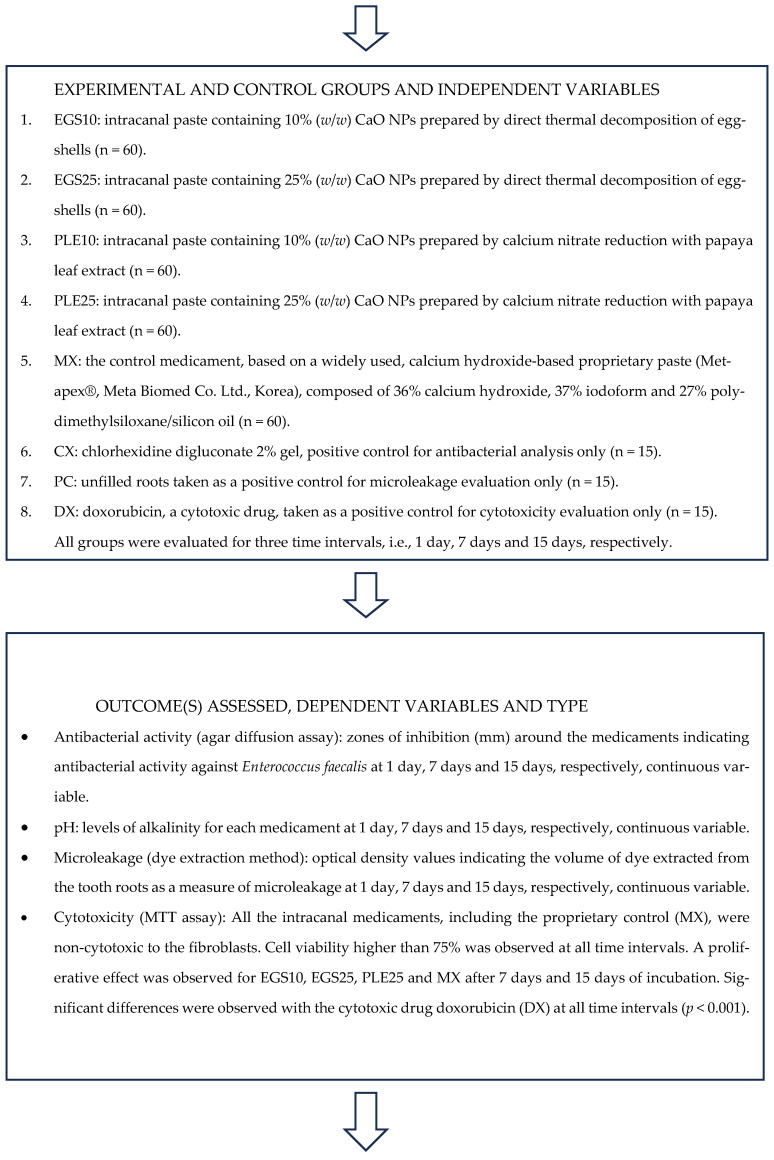

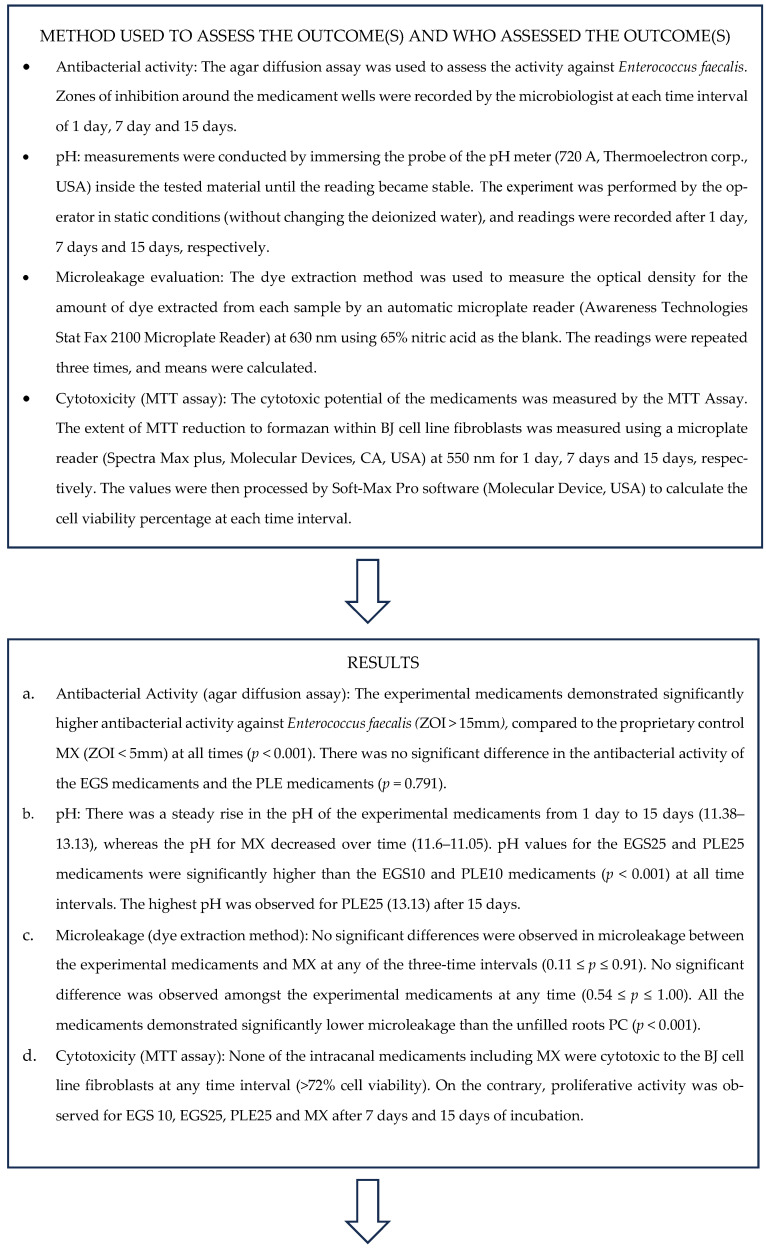

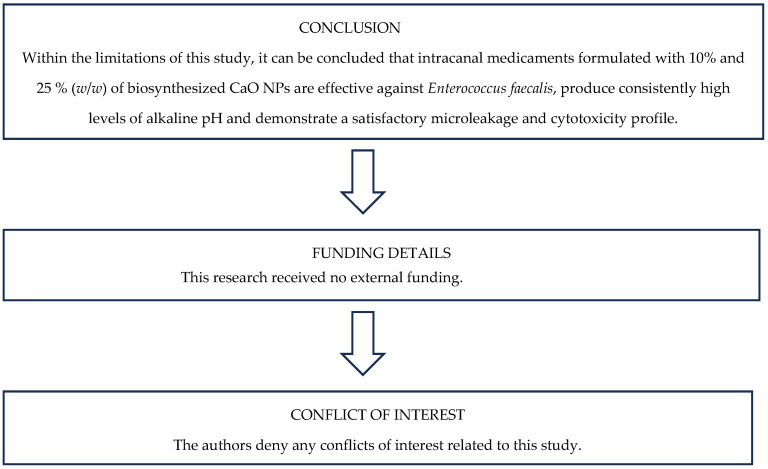
The PRILE 2021 Flowchart for the study [36,37]. From Nagendrababu, V., Murray, P.E., Ordinola-Zapata, R., Peters, O.A., Rôças, I.N., Siqueira, J.F., Jr., Priya, E., Jayaraman, J., Pulikkotil, S.J., Camilleri, J., Boutsioukis, C., Rossi-Fedele, G., Dummer, P.M.H. (2021) PRILE 2021 guidelines for reporting laboratory studies in Endodontology: a consensus-based development. International Endodontic Journal May 3. doi: 10.1111/iej.13542. https://onlinelibrary.wiley.com/doi/abs/10.1111/iej.13542 (accessed on 27 October 2024). Further details: http://pride-endodonticguidelines.org/prile (accessed on 27 October 2024).

The sample size was calculated using OpenEpi version 3.0, an open-source calculator, which ensured a 95% confidence interval and 80% statistical power by using the means and standard deviations of results from the relevant literature (Figure 6).

### 4.5. Antibacterial Activity

The antimicrobial activity of the medicaments was determined using the agar well-diffusion assay [38,39]. *Enterococcus faecalis* was procured as lyophilized pellets of ATCC 29212 Licensed Derivative^®^ (Table 5). This is a vancomycin-sensitive strain and does not exhibit resistance to gentamicin and streptomycin. It is primarily used as a control strain for antimicrobial sensitivity testing. The self-contained device, including a lyophilized microorganism pellet, reservoir of hydrating fluid and inoculating swab (KWIK-STIK™ format), was used to cultivate the organism on blood agar by gently rolling the swab over one-third of the agar plate. A sterile wire loop was then taken to streak the agar to facilitate colony isolation. This was the primary culture for *Enterococcus faecalis*. After 48 h of incubation at 35 °C, a single colony was taken from the primary culture with a sterilized wire loop, inoculated in 20 mL of Muellor Hinton broth and incubated further for 48 h, thus creating an *Enterococcus faecalis* suspension.

Characterization was carried out by isolating another colony from the agar, inoculating it in BHI broth for 24 h and then using grammes staining to confirm the presence of purple-coloured colonies of *Enterococcus faecalis*.

Later, Muellor Hinton agar medium was prepared by dissolving 38 g of agar in one litre of distilled water. This was autoclaved at 121 °C and 15 psi for about 20 min then cooled to 55 °C. Five Petri dishes were taken initially to establish the protocol, and 20 mL of the agar was poured onto each one in a biosafety cabinet. Once the medium had solidified, it was inoculated with 200 µL of *Enterococcus faecalis* suspension (0.5 McFarland, 1.5 × 10^8^ CFU/mL). The bacterial suspension was spread evenly across the entire surface by gently moving a sterilized glass spreader back and forth on the agar plate. The plate was allowed to sit for 10–15 min with the lid slightly ajar in the biosafety cabinet to let the liquid absorb into the agar. Thereafter, six wells, each of 6mm in diameter, one designated for 100 µL of medicament, were created on the agar plates.

Varying concentrations (10% to 100%) of (i) CaO NPs, (ii) commercially available calcium hydroxide (Table 5) and (iii) analytical-grade coconut oil (Table 5) were tested independently against *Enterococcus faecalis* to evaluate their antibacterial activity. The minimum effective concentrations of CaO NPs were found to be 10% and 25%, and these were selected for further evaluation. Calcium hydroxide and coconut oil did not produce any significant zones of inhibition on their own and hence were found to be inactive against *Enterococcus faecalis*.

After the establishment of the protocol, 15 agar plates inoculated with *Enterococcus faecalis* were prepared as previously described. Six wells were created containing 100 µL of each medicament, including chlorhexidine digluconate 0.2% gel (CX), which was taken as the positive control. The plates were incubated at 37 °C with 55% humidity in an aerobic atmosphere for the designated time intervals (1 day, 7 days and 15 days). Five replicates were taken for each time interval, making a total of fifteen plates for the experiment. After incubation, zones of inhibition (ZOI) surrounding the medicaments were measured in mm, indicating antibacterial activity against *Enterococcus faecalis*.

### 4.6. pH

All the groups in the study were tested for pH per ISO 23497 [40]. Calibration of the microelectrode was performed with pH 4, 7 and 10 standard solutions. An amount of 0.5 mL of each medicament was mixed in 25 mL of deionized water, and measurements were made by immersing the probe inside the tested material until the reading became stable. The probe of the pH metre (720 A, Thermoelectron corp., Waltham, MA, USA) was thoroughly washed with deionized water and wiped gently between readings. Each reading was repeated three times, and the means were calculated. The samples were stored in an incubator (BD56 Standard Incubator Avantgarde Line, Binder, Tuttlingen, Germany) at 37 °C until the next reading. The experiment was performed under static conditions after 1 day, 7 days and 15 days.

### 4.7. Microleakage Evaluation

The microleakage evaluation was performed using the dye extraction method [41]. Ninety human premolars with straight single roots, extracted for orthodontic reasons, were procured from the Oral and Maxillofacial Surgery Department, Dow University of Health Sciences, Karachi, Pakistan. The teeth were immersed in a 5.25% solution of sodium hypochlorite for 10 min, rinsed with saline and then stored in a 10% buffered formalin solution until use. The canals were prepared using ProTaper^®^ NiTi files up to F3. The teeth were decoronated at the cementoenamel junction with a root length of 15 mm (approx.). Canals were dried with paper points and filled with the corresponding intracanal medicament using a #25 lentulospiral in a slow-speed handpiece. The access cavities were sealed using glass ionomer cement (Table 5), and all root surfaces were covered with two layers of nail varnish, sparing the apical 2 mm. The filled roots were then immersed in 2% methylene blue solution and incubated for 1 day, 7 days and 15 days, respectively. Unfilled teeth were taken as the positive control. At the end of the incubation period, the roots were removed from the dye, washed with copious amounts of water and nail varnish removed with acrylic finishing bur in a slow-speed handpiece. Each root was placed in a vial containing 1000 μL of 65% nitric acid (Table 5) for 3 days. Later, the roots were removed from the vials and centrifuged at 10,000 rpm for 5 min. The supernatant from each vial was transferred to a 96-well plate, and optical density was read by an automatic microplate reader (Awareness Technologies Stat Fax 2100 Microplate Reader) at 630 nm, using 65% nitric acid as the blank. The readings were repeated three times, and means were calculated.

### 4.8. Cytotoxicity Testing

Cytotoxicity of the compounds was determined by the 3-[4, 5-dimethylthiazole-2-yl]-2, 5-diphenyl-tetrazolium bromide (MTT) assay [2]. BJ human fibroblast cells (ATCC # CRL-2522) were cultured in Dulbecco’s Modified Eagle Medium (DMEM) with 10% fetal bovine serum, 100 IU/mL of penicillin, 100 µg/mL of streptomycin and 2 mM L-glutamine in 75 cm^2^ flasks, incubated in 5% CO_2_ at 37 °C. Exponentially growing cells were trypsinized, counted with a hemocytometer and diluted with medium to a concentration of 9 × 10^4^/mL. Cells (100 µL/well) were introduced into a 96-well plate. After overnight incubation, the old medium was removed, and 200 µL of fresh medium was added to 30 µM of the intracanal medicaments. MTT (0.5 mg/mL) was added to each well and incubated further for 3 h. After incubation, the MTT was removed, and formazan crystals were dissolved in 100 µL of dimethyl sulfoxide (DMSO). The extent of MTT reduction to formazan within cells was calculated by measuring the absorbance at 550 nm using a microplate reader (Spectra Max Plus, Molecular Devices, San Jose, CA, USA). The experiment was carried out in triplicates after 1 day, 7 days and 15 days of incubation. Values were processed (Soft-Max Pro 7 software, Molecular Devices, San Jose, CA, USA), and the results were expressed as % cell viability.

### 4.9. Statistical Analysis

MS Excel (Redmond, WA, USA: Microsoft Corp. 2016) and SPSS version 23 (Armonk, NY, USA: IBM Corp. 2015) were used to compile data and conduct statistical analysis. The means and standard deviations for all the outcome variables were calculated. Three-way ANOVA was conducted for antibacterial, microleakage and cytotoxicity analyses; repeated-measures ANOVA was carried out for pH analysis. Post hoc analysis was performed using LSD. A *p*-value < 0.05 was considered significant.

## 5. Conclusions

Within the limitations of this study, it can be concluded that intracanal medicaments formulated with 10% and 25 % (*w*/*w*) of biosynthesized CaO NPs are effective against *Enterococcus faecalis*, produce consistently high levels of alkaline pH and demonstrate a satisfactory microleakage and cytotoxicity profile.

## Figures and Tables

**Table 1:** Antibacterial activity observed for the intracanal medicaments at different time intervals.

Group	Zone of Inhibition (mm ± SD)		
	**24 h**	**7 Days**	**15 Days**
EGS10	16.40 ± 2.48 ^aA^	15.40 ± 1.78 ^aA^	16.60 ± 2.63 ^aA^
EGS25	17.80 ± 4.25 ^aA^	15.70 ± 2.86 ^aA^	17.60 ± 2.63 ^aA^
PLE10	16.60 ± 5.59 ^aA^	16.20 ± 2.58 ^aA^	17.30 ± 1.92 ^aA^
PLE25	16.50 ± 3.46 ^aA^	16.70 ± 1.03 ^acA^	19.60 ± 2.30 ^aA^
MX	4.10 ± 3.74 ^bA^	1.70 ± 3.80 ^bAB^	0.00 ± 0.00 ^bB^
CX	19.70 ± 1.03 ^aC^	20.00 ± 1.27 ^cC^	19.40 ± 0.65 ^aC^

*p*-value ≤ 0.05 was considered significant; different superscript letters indicate significant differences in post hoc analysis; small letters indicate column-wise comparison; capital letters indicate row-wise comparison.

**Table 2:** pH values for the intracanal medicaments at different time intervals.

Group	pH ± SD		
	**24 h**	**7 Days**	**15 Days**
EGS10	11.38 ± 0.76 ^aA^	11.90 ± 0.78 ^aB^	12.71 ± 0.47 ^aC^
EGS25	11.89 ± 0.56 ^bA^	12.40 ± 0.48 ^bB^	12.99 ± 0.39 ^bC^
PLE10	11.61 ± 0.78 ^cA^	12.02 ± 0.72 ^aB^	12.73 ± 0.44 ^aC^
PLE25	12.02 ± 0.59 ^bA^	12.40 ± 0.47 ^bB^	13.13 ± 0.35 ^bC^
MX	11.60 ± 0.14 ^cA^	11.95 ± 0.13 ^aB^	11.05 ± 0.18 ^cC^

*p*-value ≤ 0.05 was considered significant; different superscript letters indicate significant differences in post hoc analysis; small letters indicate column-wise comparison; capital letters indicate row-wise comparison.

**Table 3:** Microleakage observed for the intracanal medicaments at different time intervals, indicated by optical density measurement after dye extraction.

Group	Optical Density ± SD		
	**24 h**	**7 Days**	**15 Days**
EGS10	0.29 ± 0.08 ^aA^	0.38 ± 0.03 ^aB^	0.75 ± 0.37 ^aC^
EGS25	0.46 ± 0.20 ^aA^	0.49 ± 0.17 ^aA^	0.75 ± 0.24 ^aB^
PLE10	0.39 ± 0.16 ^aA^	0.41 ± 0.21 ^aA^	0.63 ± 0.03 ^aB^
PLE25	0.45 ± 0.19 ^aA^	0.46 ± 0.22 ^aA^	0.74 ± 0.16 ^aB^
MX	0.40 ± 0.14 ^aA^	0.64 ± 0.05 ^bB^	0.76 ± 0.03 ^aC^
PC	0.98 ± 0.52 ^bA^	1.00 ± 0.55 ^cA^	1.46 ± 0.10 ^bB^

*p*-value ≤ 0.05 was considered significant; different superscript letters indicate significant differences in post hoc analysis; small letters indicate column-wise comparison; capital letters indicate row-wise comparison.

**Table 4:** Cytotoxicity results presented as cell viability percentage of fibroblasts at different time intervals in the MTT Assay.

Group	Cell Viability ± SD		
	**24 h**	**7 Days**	**15 Days**
EGS10	98.3 ± 0.10 ^aA^	99.3 ± 0.06 ^aB^	101.1 ± 0.12 ^aC^
EGS25	98.4 ± 0.10 ^aA^	100.3 ± 0.03 ^bB^	72.2 ± 0.14 ^bC^
PLE10	98.7 ± 0.05 ^aA^	97.5 ± 0.28 ^cB^	98.7 ± 0.03 ^cA^
PLE25	97.0 ± 0.18 ^bA^	110.2 ± 0.31 ^dB^	100.9 ± 0.03 ^aC^
MX	93.3 ± 0.23 ^cA^	105.4 ± 0.16 ^eB^	114.8 ± 0.06 ^dC^
DX	14.17 ± 0.05 ^dA^	13.68 ± 0.15 ^fA^	14.02 ± 0.11 ^eA^

*p*-value ≤ 0.05 was considered significant; different superscript letters indicate significant differences in post hoc analysis; small letters indicate column-wise comparison; capital letters indicate row-wise comparison; values greater than 100 indicate proliferative activity.

**Table 5:** The primary materials employed in this study.

Material	Specifications
Eggshells	Regular broiler chicken eggshell household waste
*Carica papaya* leaves	Garrison Nursery, Malir Cantt, Karachi, Pakistan
Calcium nitrate tetrahydrate powder	Lot# COO43RF1, CAS# 13477-34-4, DaeJung Chemicals and Metals Co., Ltd., Gyeonggi, Republic of Korea
Sodium hydroxide pellets	Lot# SZB9320A, CAS# 1310-73-2, Sigma-Aldrich, Praha 4-Nusle, Czech Republic
Coconut oil	Lot# LRAC6922, CAS# 8001-31-8, 46,949 Coconut Oil analytical standard, Sigma Aldrich, St. Louis, MO, USA
Calcium Hydroxide powder	Lot# F2254871, CAS# 1305-62-0, ‘AnalaR’, BDH Laboratory Supplies, Middlesex, England; VWR International, Radnor, PA, USA.
*Enterococcus faecalis*	ATCC 29212 Licensed Derivative^®^, Lot#366-407-2, Microbiologics^®^ Inc., St Cloud, MN, USA
Metapex Plus^®^	Meta Biomed Co., Ltd., Cheongju-si, Republic of Korea, Lot#MXP2111171, MXP2208171.
Nitric acid	Lot#K50746856, CAS#1.00456.1000, Emsure^®^ ISO Nitric acid, Merck, Darmstadt, Germany
Glass ionomer cement	Lot#2102061, GC Gold Label Glass Ionomer, Universal Restorative, GC, Tokyo, Japan

## Data Availability

The datasets used and/or analyzed during the current study are available from the corresponding author upon reasonable request.
